# A Stepwise Guide to Performing Shoulder Ultrasound: The Acromio-Clavicular Joint, Biceps, Subscapularis, Impingement, Supraspinatus Protocol

**DOI:** 10.7759/cureus.18354

**Published:** 2021-09-28

**Authors:** Lauren Ann J Selame, Bridget Matsas, Benjamin Krauss, Andrew J Goldsmith, Hamid Shokoohi

**Affiliations:** 1 Emergency Department, Brigham and Women's Hospital, Boston, USA; 2 Harvard University, Harvard Medical School, Boston, USA; 3 Renaissance School of Medicine, Stony Brook University, Stony Brook, USA; 4 Emergency Department, Massachusetts General Hospital, Boston, USA

**Keywords:** tendon injury, shoulder dislocation, subacromial impingement, rotator cuff, shoulder pain, ultrasound

## Abstract

Shoulder pain is a common and painful patient condition. Unfortunately, diagnostic imaging of shoulder pain in the emergency department (ED) is often limited to radiography. While diagnostic for fractures and dislocations, drawbacks of radiography include time delays and non-diagnostic imaging in the case of rotator cuff pathology. While bedside ultrasound has been incorporated into many procedural and diagnostic applications in the ED, its use for musculoskeletal complaints and specifically shoulder pain is infrequent among ED clinicians. The incorporation of shoulder ultrasound in the ED may improve diagnostic certainty while decreasing time to diagnosis and treatment, yielding patient and health system benefits. Herein, we present the ABSIS (Acromio-clavicular joint, Biceps, Subscapularis, Impingement, Supraspinatus) Protocol for performing bedside ultrasound of the shoulder including the rotator cuff and bony anatomy.

## Introduction

Timely and accurate diagnosis of shoulder pathology can be challenging in the emergency department (ED). Acute shoulder pain and injuries are among the most common upper extremity complaints of ED patients [1].

While radiographs are suggested for the initial workup of shoulder pain, their utility is often limited to bony pathology [2,3]. Magnetic resonance imaging (MRI) and magnetic resonance arthrography (MRA), have high accuracy and reproducibility in the diagnosis of tendon, ligamental, and muscular injuries; however, although patient satisfaction may improve with definitive diagnosis and treatment plan, these imaging modalities are costly and poorly accessible in many locations [4-6]. The utility of ultrasound in diagnosing rotator cuff tears has been well-described, with similar accuracy to MRI [4,7,8].

Despite high accuracy and use in specialties such as musculoskeletal radiology, rheumatology, physical medicine and rehabilitation, and sports medicine, shoulder ultrasound is not routinely used in the ED. Ultrasound offers a less expensive and more accessible alternative to evaluate shoulder pain and inform patient management. In this guide, we present a stepwise protocol for performing and interpreting ultrasound of the shoulder, the ABSIS (Acromio-clavicular joint, Biceps, Subscapularis, Impingement, Supraspinatus) Protocol.

## Technical report

The probe marker should be oriented cephalad or toward the patient’s right throughout the ABSIS Protocol. As some conventions call for medial probe marker orientation, image laterality should be labeled for clarity. A video guide to this protocol with accompanying normal and pathologic images is presented (Video 1).

**Video 1:** A Stepwise Guide to Performing Shoulder Ultrasound (A Simple Guide to Shoulder Ultrasound) In this video, a systematic ultrasound examination of the shoulder including evaluation of bony anatomy and tendons is performed through the ABSIS (Acromio-clavicular joint, Biceps, Subscapularis, Impingement, Supraspinatus) Protocol. Normal anatomy and acute pathology including dislocations, fractures, and tendon tears are presented.

Table 1 outlines the location, function, and ultrasound anatomy of the tendons evaluated in the ABSIS Protocol.

**Table 1:** Tendon Anatomy, Function, and Ultrasound Characteristics

Muscle	Origin	Insertion	Function	Arm Position	Ultrasound Characteristics
Biceps brachii	Long head: supraglenoid tubercle. Short head: coracoid process	Radial tuberosity and fascia of forearm	Forearm flexion and supination	Arm flexion	The long head of the biceps tendon traverses within the bicipital groove. Shoulder effusions may lead to fluid around this tendon.
Subscapularis	Scapula (subscapular fossa)	Humerus (lesser tuberosity)	Internal rotation, adduction	External rotation	Myotendinous structure with the appearance of multiple tendons within the muscle.
Supraspinatus	Scapula (supraspinous fossa)	Humerus (greater tuberosity, superior facet)	Abduction (initial 0 to 15 degrees)	Crass positioning or internal rotation	Standard feather-like tendinous appearance with anisotropy
Infraspinatus	Scapula (infraspinous fossa)	Humerus (greater tuberosity, middle facet)	External rotation	Overlies posterior glenohumeral joint	Standard feather-like tendinous appearance with anisotropy

The acromion-clavicular joint

Arm Position

Begin the examination with the patient sitting with the symptomatic arm in 90 degrees of flexion with the forearm supinated and resting on the ipsilateral thigh. As in the remainder of the protocol, if this is not possible a position of comfort should be used.

Key Anatomy 

The acromio-clavicular (AC) joint is formed by the acromion process and distal clavicle, which are attached by the AC ligament and encompassed by the AC joint capsule. The trapezius muscle runs over the superior aspect of the AC joint, while the supraspinatus runs inferior to the AC joint.

There are limited studies describing the normal length, width, and height of the AC joint on ultrasound, and these measurements may be dependent on multiple factors such as age and habitus [9-11]. Therefore, a focused evaluation of the horizontal alignment of the clavicle and acromion process is critical to identifying AC joint pathology. Comparison with the contralateral, asymptomatic side may illuminate significant differences between the AC joints and is therefore useful in diagnosing AC joint pathology [10].

Scan Technique

**Figure 1:**
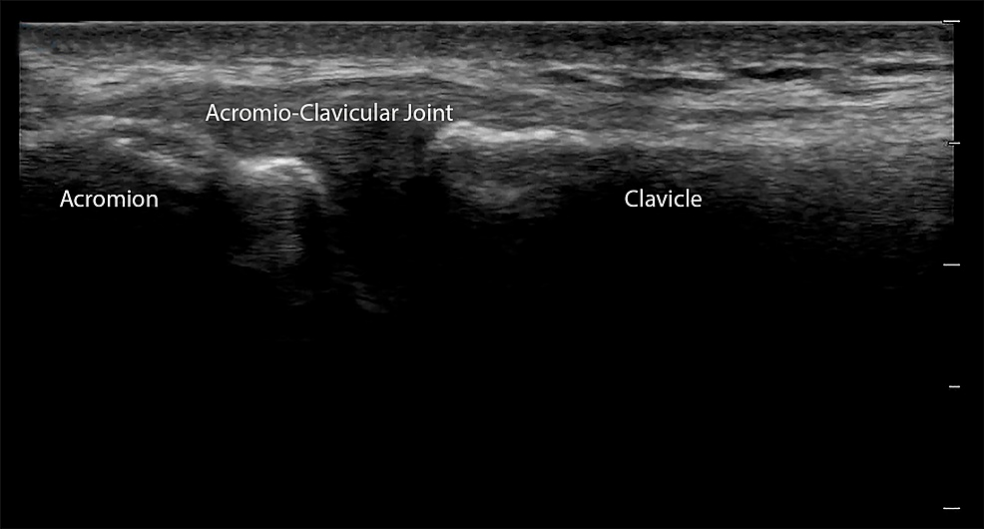
The Acromio-Clavicular Joint Longitudinal view of the acromio-clavicular joint space. The hypoechoic space between the hyperechoic lateral clavicle and the medial edge of the acromion is the acromio-clavicular joint space.

Using a high-frequency linear probe, evaluate the AC joint in transverse orientation with the probe marker facing the patient’s right. Begin by palpating the clavicle, then following it laterally (via palpation or ultrasound) until you reach the AC joint. The AC joint normally appears as a V-shaped hypoechoic to an anechoic area between the lateral clavicle and the acromion (Figure 1).

Perform dynamic evaluation of AC joint by gently pulling arm downward to confirm laxity and expansion of the joint on axial load.

Pathologies

Rheumatologic disorders including lupus and gout may cause synovial enlargement or crystal formation. Traumatic AC joint separations are demonstrated by increased horizontal play of the AC joint and downward displacement of the lateral portion following AC ligament injury [10]. Radiographic AC separations are categorized using Rockwood classifications but an ultrasound classification is yet to be determined [12,13].

The long head of the biceps tendon

Arm Position

Symptomatic arm in 90 degrees of flexion with the forearm supinated and resting on ipsilateral thigh.

Key Anatomy

The long head of the biceps tendon travels between the lesser and greater tuberosities and beneath the transverse humeral ligament. Because the biceps tendon origin site is within the gleno-humeral joint, a shoulder effusion may lead to the development of hypoechoic fluid surrounding the biceps tendon. This can be misinterpreted as biceps pathology.

Scan Technique

**Figure 2:**
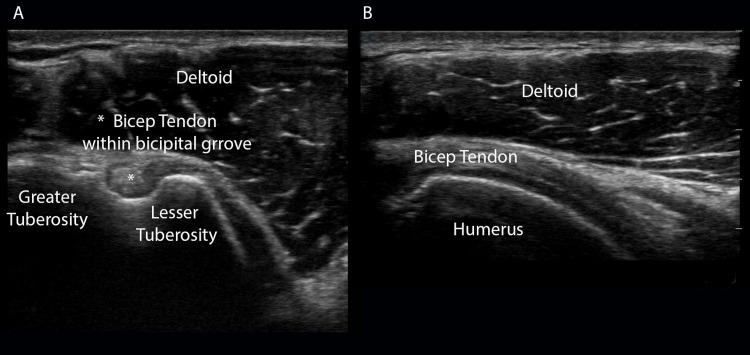
Long Head of the Biceps Tendon (A) Long head of the biceps tendon in transverse as it travels through the bicipital groove between greater and lesser tuberosities and (B) 90-degree clockwise rotation of the ultrasound probe will reveal a long axis view of the bicep tendon.

From the AC joint, palpate or track the probe inferiorly until you locate the bicipital groove, situated between the greater and lesser tuberosities of the humeral head. The long head of the biceps tendon travels within this groove. Slide the probe caudad to examine the tendon in its entirety in this transverse orientation. With the biceps tendon in the center of the screen, rotate the probe 90 degrees - so the probe is oriented cephalad - and examine the tendon in longitudinal view (Figure 2A-2B).

Perform dynamic evaluation by having the patient supinate and flex their arm.

Pathologies

Acute tendon injuries and bicipital tendinopathies can be diagnosed via the identification of tendon disruptions, retractions, surrounding fluid, and irregularities or deposits along the tendon.

Subscapularis

Arm Position

With their symptomatic arm in 90 degrees of flexion with forearm supinated and resting on ipsilateral thigh next have the patient externally rotate their arm 90 degrees or as far as is comfortable.

Key Anatomy

**Figure 3:**
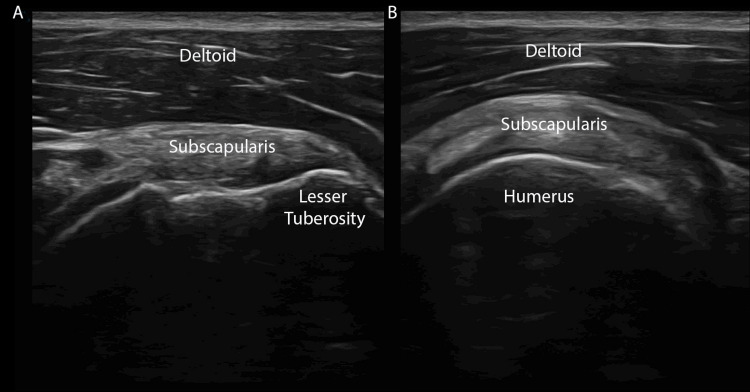
Subscapularis (A) Long axis view of the subscapular tendon. The bird’s beak appearance as it attaches to the lesser tuberosity is demonstrated. (B) Rotation of the ultrasound probe 90 degrees clockwise will yield a transverse view of the subscapularis and the subscapularis’ myotendinous structure with characteristic fibular tendinous anisotropic bundles.

The subscapularis originates from the subscapular fossa and inserts on the lesser tuberosity, medial to the long head of the biceps tendon. A unique characteristic of the subscapularis tendon is its myotendinous structure with characteristic fibular tendinous bundles exhibiting anisotropy within an anechoic feathery surrounding. This appearance can be misinterpreted as pathologic (e.g., tendon rupture), given the physiologic anechoic portions of the myotendinous structure (Figure 3A).

Scan Technique

With a transverse view of the bicipital groove, have the patient externally rotate their arm. With this movement, note the appearance of the “bird’s beak” shaped subscapularis tendon where it inserts into the lesser tuberosity. Evaluate the tendon in longitudinal view, then rotate the probe 90 degrees so the marker faces cephalad, to examine the tendon in its transverse plane (Figure 3A-3B).

Pathologies

Tendon tears, ruptures, and tendinopathies.

The subacromial space (impingement)

Arm Position

Symptomatic arm in a position of comfort.

Key Anatomy

The subacromial space lies beneath the AC joint and coracoacromial ligament, and it encompasses the supraspinatus tendon and subacromial bursa. The impingement interval is the area between the undersurface of the acromion and the superior aspect of the humerus. The supraspinatus tendon runs through the impingement interval and is at risk of being compressed during abduction [14]. Impingement can be caused by extrinsic compression or loss of function of the rotator cuff (which depresses the humeral head), as the unopposed deltoid abducts the humerus, decreasing the small space between the acromion and humerus. The subdeltoid and subacromial bursas may also develop inflammation and cause symptomatic shoulder impingement.

Scan Technique

**Figure 4:**
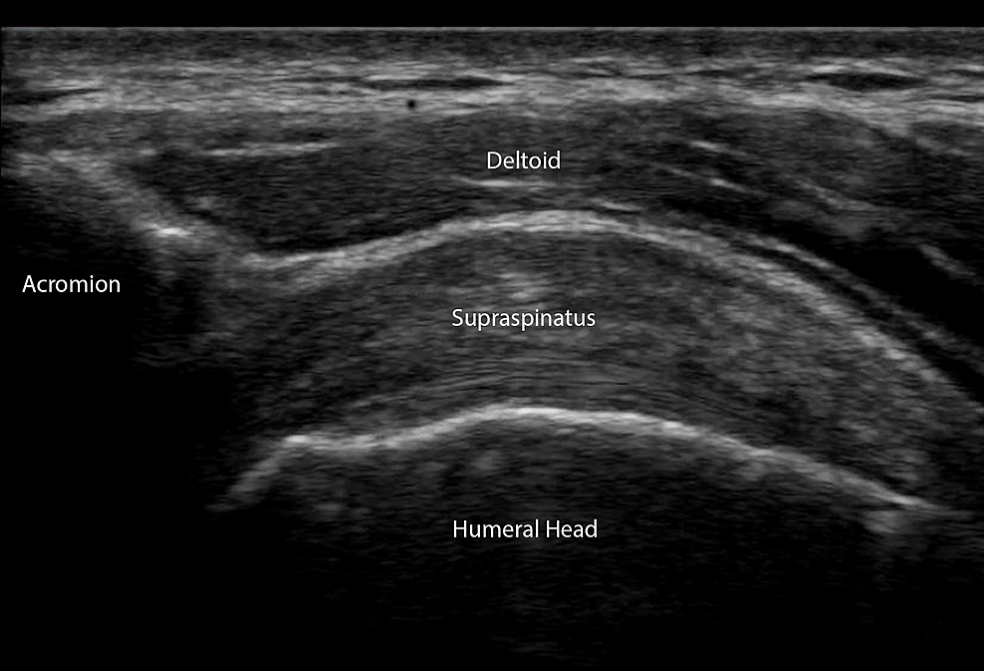
The Subacromial Space Longitudinal view of the supraspinatus as it traverses the subacromial space between the acromion process and humeral head. The subdeltoid and subacromial bursas may become inflamed and lead to impingement syndrome.

With the probe at the AC joint and the marker facing the patient’s right, slide the probe laterally until the clavicle is off the screen and the acromion process, humeral head, and supraspinatus tendon are visualized (Figure 4).

Use dynamic evaluation with shoulder abduction to assess for mechanical impingement of the supraspinatus and/or pain. If the patient reports pain that correlates with movement through this arc, yet you do not visualize pathology, the patient may still have symptomatic subacromial impingement [15].

Pathologies

Supraspinatus tear or rupture, mechanical impingement of the supraspinatus (e.g., spurs, osteophytes, avulsion fracture), and any etiologies of subacromial soft tissue swelling (e.g., rotator cuff tendonitis, rotator cuff contusion, bursitis) which may cause impingement or pain.

Supraspinatus

Arm Position

**Figure 5:**
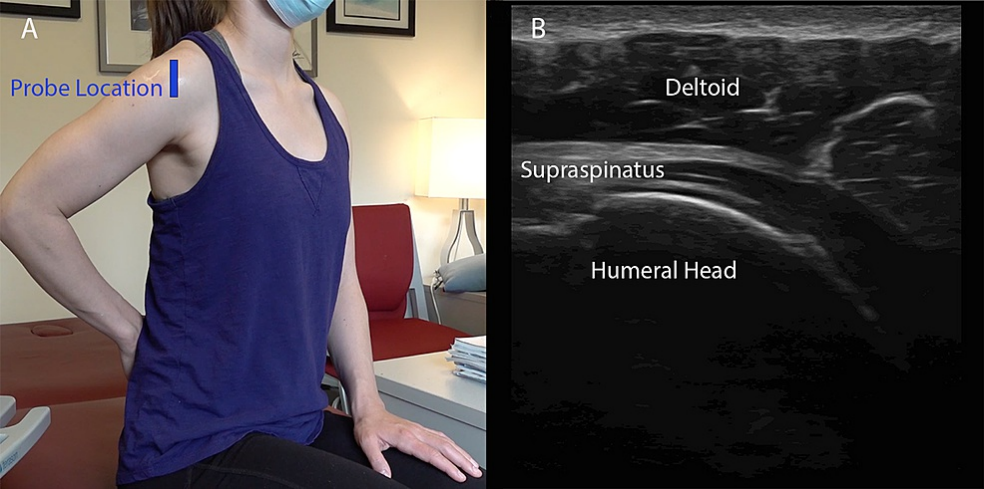
Supraspinatus (A) Demonstration of Crass positioning which will best expose the supraspinatus tendon. The dorsal aspect of the hand faces the patient’s lower back and the location of the ultrasound probe in order to obtain a long-axis view of the supraspinatus is shown. (B) The acquired long-axis view of the supraspinatus tendon. The probe may be rotated 90 degrees clockwise to obtain a transverse view of the tendon.

Crass positioning is preferred (Figure 5A). If this is not possible for the patient, with their arm in extension, have them perform internal rotation of the shoulder.

Key Anatomy

The supraspinatus muscle travels from the superior aspect of the scapular spine, right beneath the AC joint, and inserts on the greater tuberosity of the humerus.

Scan Technique

Place the probe - with the marker facing cephalad, but angled parallel to the humerus - below the level of the acromion, to visualize the supraspinatus tendon on a long axis (Figure 5B).

Rotate the probe - so the marker faces rightward - and examine the tendon in a transverse orientation.

Pathologies

Tendon partial or complete tear, tendinopathy, impingement (see The Subacromial Space).

Glenohumeral joint

To be completed if there is clinical concern for dislocation or shoulder effusion.

Arm Position

Symptomatic arm in a position of comfort.

Key Anatomy

The glenohumeral joint consists of the humeral head, the glenoid fossa of the scapula, and the surrounding synovial membrane. The long head of the biceps brachii travels through the glenohumeral joint space.

Scan Technique

**Figure 6:**
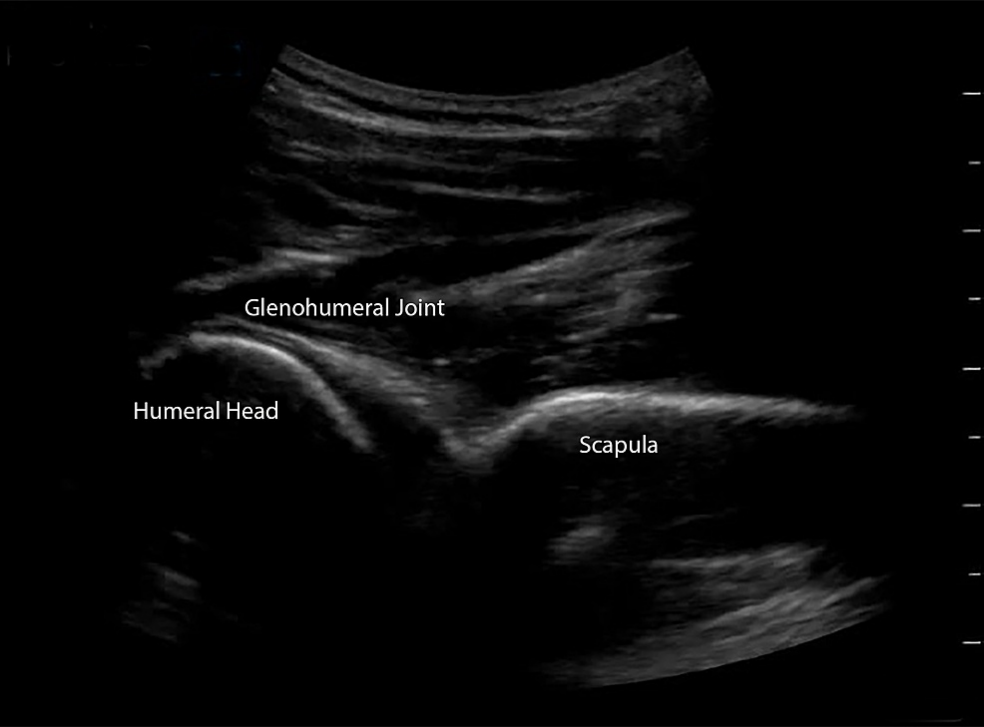
Glenohumeral Joint In this posterior view of the glenohumeral joint, the ultrasound probe is oriented transversely at the level of the scapular spine. A posterior view of the glenohumeral joint demonstrates proper horizontal alignment of the glenoid fossa of the scapula and the humeral head. The humeral head may be directly visualized moving within the glenoid fossa to confirm alignment. Lack of horizontal alignment of the scapula and the humeral head is pathologic and indicative of dislocation. The joint effusion may also be appreciated in this location.

For this examination, the curvilinear probe may be preferable, depending on body habitus. In the posterior aspect place the probe, oriented transversely, below the scapular spine at the level of the posterior glenohumeral joint. Examine the posterior humeral head and scapula to evaluate for horizontal alignment of the joint, and for potential effusion (Figure 6).

**Figure 7:**
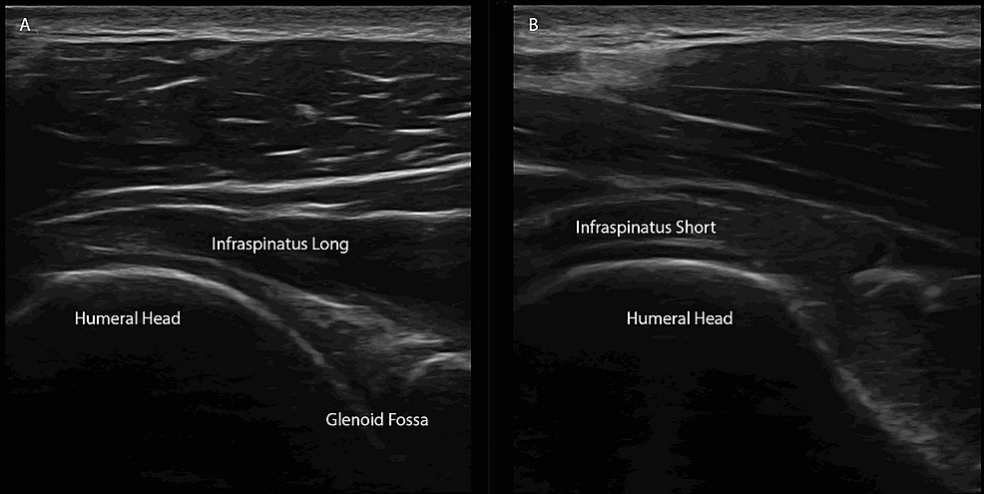
Infraspinatus (A) In this posterior view at the level of the glenohumeral joint beneath the scapular spine, the infraspinatus tendon may be visualized traversing above the glenohumeral joint. (B) The short view of the infraspinatus tendon is visualized with the ultrasound probe in the longitudinal orientation.

Lack of horizontal alignment suggests shoulder dislocation. In this view of the glenohumeral joint, the infraspinatus tendon traverses above the glenohumeral joint and may be evaluated in longitudinal and transverse planes (Figure 7A-7B).

Dynamically evaluate the joint during shoulder external rotation.

Pathologies

Glenohumeral joint dislocation or joint effusion.

Proximal humerus

To be completed if there are clinical concerns for proximal humerus fracture.

Arm Position

Symptomatic arm in a position of comfort.

Key Anatomy

Fractures may appear as cortical disruptions, step-offs, or displaced fragments. Acute fractures generally have overlying hematomas and tenderness. It is important to note that rotator cuff tendinopathy, inflammatory disorders, and degenerative disorders can cause surface erosions that may appear as cortical disruptions. The evaluation of cortical disruptions should be interpreted in the setting of the patient’s clinical presentation and mechanism of injury. Avulsion fractures of the greater tuberosity may appear as elevated bony fragments and can be mistaken for calcific tendonitis [16].

Scan Technique

**Figure 8:**
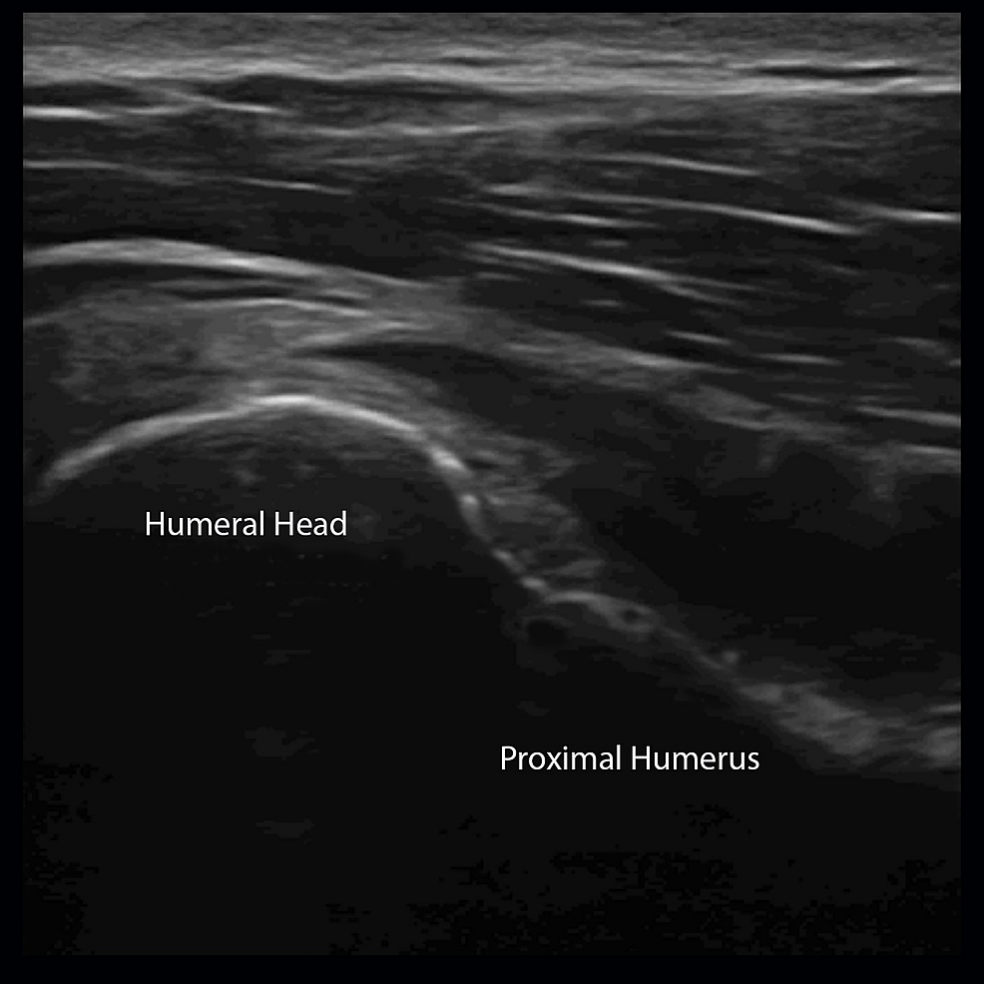
Proximal Humerus A coronal view of the humerus is obtained with the ultrasound probe with marker-oriented cephalad in order to visualize the humeral head and proximal humerus.

Place the probe - with the marker-oriented cephalad - on the lateral margin of the proximal humerus. Locate the humeral head, which should appear lightbulb-shaped with even contours (Figure 8).

Fan through the humeral head, paying close attention to areas of discomfort.

Pathologies

Proximal humerus fracture, greater tuberosity avulsion fracture.

Infraspinatus and teres minor

Isolated infraspinatus and teres minor tendon injuries are uncommon. Evaluation of the infraspinatus tendon may be performed during the examination of the posterior glenohumeral joint as previously described. However, evaluation of the teres minor tendon is not included in this protocol.

## Discussion

The ABSIS protocol guides the clinician through a stepwise shoulder ultrasound evaluation for undifferentiated shoulder complaints. Tailored examinations include careful evaluation of the rotator cuff and/or surrounding bony anatomy relevant to the patient’s presentation. As with all diagnostics tests, taking a careful history and physical examination should guide appropriate incorporation of the ABSIS Protocol, inform the clinical impression, and guide patient management in order to improve diagnostic certainty.

Important branch points when incorporating the ABSIS protocol include consideration of suspicion for shoulder dislocation, fracture-dislocation, fracture, rotator cuff, or AC separation. If the provider’s primary clinical concern is for simple dislocation, a shoulder examination may start with a pre-reduction gleno-humeral joint evaluation and end with a post-reduction gleno-humeral joint evaluation. However, any confirmation of dislocation with concern for concomitant fractures, such as proximal humerus fracture, should be combined with a direct ultrasound evaluation of the proximal humerus. Use of this practice may expedite definitive management and orthopedic consultation in cases that are heading down the route toward consultation such as in fracture-dislocation. On the other hand, for patients with history and physical examinations which do not suggest fracture or shoulder dislocation, a stepwise shoulder evaluation may flow directly through each step of the ABSIS Protocol.

As the access to imaging modalities such as MRI and MRA is limited by geography, wait times, and cost, the ability for clinicians to perform real-time shoulder ultrasounds may greatly improve patient experience, expedite diagnosis and management, and decrease healthcare expenditures [5,17]. As MRI is over-utilized in some settings, this practice puts unnecessary strain on already overburdened healthcare systems in some locations, while the ability to diagnose and treat patients is limited in other settings given poor access to MRI [17]. This simultaneous over-utilization and poor access to MRI may be overcome through focused training and implementation of the ABSIS protocol.

Limitations

There are important limitations of the ABSIS protocol which must be considered. The accuracy of ultrasound is operator-dependent, limited by learning curves, and its sensitivity varies by disease severity [18]. For example, partial tendon tears are more likely to be missed on ultrasound than complete tendon tears, although there is evidence to suggest that ultrasound is still more sensitive and specific than MRI for detecting partial tears [7,19]. While there is research to support the moderate to substantial agreement of shoulder ultrasound and MRA as interpreted by radiologists, there is a limited investigation of shoulder ultrasound including the rotator cuff performed by other specialties [7]. Given the lower prevalence of posterior shoulder dislocations in comparison to anterior dislocations, there exists limited research on the accuracy of ultrasound for the diagnosis of posterior shoulder dislocation [20]. Although there is evidence to suggest that ultrasound may be a suitable singular imaging modality for anterior shoulder dislocations, evidence to support the singular use of ultrasound without radiography in suspected upper limb fractures or AC joint separation is lacking [11,20,21].

## Conclusions

The accuracy of shoulder ultrasound has been demonstrated in comparison to advanced imaging with MRI, but its implementation in settings such as the ED has been limited. While performance and interpretation of bedside ultrasound are user-dependent, focused training and use of the ABSIS protocol for shoulder ultrasound may improve diagnostic certainty and expedite definitive patient management for patients with shoulder complaints. The next steps include monitoring learning curves and research investigations on clinical impacts and patient satisfaction with the integration of the ABSIS protocol in clinical medicine.
